# Evaluating the quality of technology integration across seven European countries with the ICAP Technology Scale

**DOI:** 10.1007/s40692-024-00341-y

**Published:** 2024-12-18

**Authors:** Mirjana Maričić, Branko Anđić, Filiz Mumcu, Lukáš Rokos, Jan Vondruška, Robert Weinhandl, Zsolt Lavicza, Andreja Špernjak

**Affiliations:** 1 Department of Sciences and Management in Education, School of Education, University of Novi Sad, Podgorička 4, 25000 Sombor, Republic of Serbia; 2 Department of STEM Education, School of Education, Johannes Kepler University, Altenberger Straße 69, Standort, Science Park 4, 4040 Linz, Austria; 3 Department of Computer Education and Instructional Technologies, Faculty of Education, Manisa Celal Bayar University, Demirci, 45900 Turkey; 4 Department of Biology, Faculty of Education, University of South Bohemia, Jeronýmova 10, České Budějovice, Czech Republic; 5 Faculty of Natural Sciences and Mathematics, University of Maribor, Koroška Cesta 160, 2000 Maribor, Slovenia

**Keywords:** Cognitive engagement, ICAP-TS, Learning activities, Technology integration

## Abstract

Interactive, constructive, active and passive technology scale (ICAP-TS) is a relatively new developed instrument representing an essential literature need. Through this cross-cultural study, we strived to accomplish a three-fold aim. Firstly, we aspired to verify the scale's construct validity and reliability on a large sample of teachers across seven European countries. Secondly, we aimed to evaluate the quality of technology integration (TI) on this heterogeneous sample with the ICAP-TS to explore for which learning activities teachers use technologies, within which ICAP learning/engaging modes and how often. Thirdly, we strived to examine relationships between the different technology types (TT) and TI in ICAP learning modes. The research involved 2277 primary and lower secondary school teachers. Confirmatory factor analysis revealed that the internal structure of ICAP-TS corresponds well to the overall sample, but for some countries, the model fit should be further refined. Exploratory factor analysis extracted two basic components of TT—*passive* and *active*. On a general level teachers most often integrate technology into passive learning mode with the usage of passive TT, but if these results are observed within each country separately, this is not the case everywhere. Passive TT predicts TI into passive, active, and constructive modes more, while active TT predicts TI into interactive, constructive and active modes. This study has several implications. For example future research topics can include reviewing, revising, or adding new items to the ICAP-TS related to ICAP theory to improve its validity. Other recommendations are stated in the discussion.

## Introduction

The quality of technology integration (TI) highly depends on how digital technologies are used to cognitively engage students and involve them in various learning activities, not only on the degree of its frequency in teaching nor on the type of technology that has been integrated (Antonietti et al., 2023a; Anđić et al., 2024a; Deepika et al., 2022; Maričić & Lavicza, 2024; Radulović et al., 2022; Wekerle et al., 2022; Xianhan et al., 2022). Although this issue has been widely researched and emphasized in the literature (Fütterer et al., 2022), these studies utilised either somewhat simplified or quite complex techno-centric scales for assessing TI (Antonietti et al., 2023a). Their focus is on examining the level of TI frequency, acceptance of integration, and use of different digital tools without direct revealing information about how technology is integrated into teaching and within which activities (Antonietti et al., 2023a; Dwivedi et al., 2020; Schmitz et al., 2022). Given that the quality of TI, among other things (such as teacher support and classroom management), is reflected in the degree to which it is used to transform and redefine learning activities to ensure and support the cognitive activation of students (Backfisch et al., 2021), it is very important to gain insight into how technology is integrated into teaching and within what kinds of activities. This process requires the usage of a theoretically based, reliable, and valid measurement tool that can assess different types of teaching and learning activities in which technology is integrated, as well as whether technology is used as a substitute for traditional teaching style or to support deeper cognitive processes (Consoli et al., 2023). At the beginning of 2023, Antonietti et al. (2023a) developed a theoretically-based instrument with a scale to successfully measure the way teachers integrate technology into various teaching and learning activities—the so-called ICAP-TS (Interactive, Constructive, Active, and Passive—Technology Scale), which is based on the four-level activity framework (from passive to interactive) by Chi and Wylie (2014). Based on the recommendations derived from previous research, as well as the originality of our study (explained in the Present Study section), we formulated a three-fold aim. Firstly, we aspired to verify the scale's construct validity and reliability on a large sample of teachers across seven European countries. Secondly, we aimed to evaluate the quality of TI on this heterogeneous sample with the ICAP-TS to explore for which learning activities teachers use technologies, within which ICAP learning modes, and how often. Thirdly we strived to examine relationships between the different technology types (TT) and TI in ICAP learning modes. To reach these aims, we need to introduce our developed ICAP-TS grounded in ICAP theory in the upcoming sections.

## Grounding in the ICAP theory

**Fig. 1:**
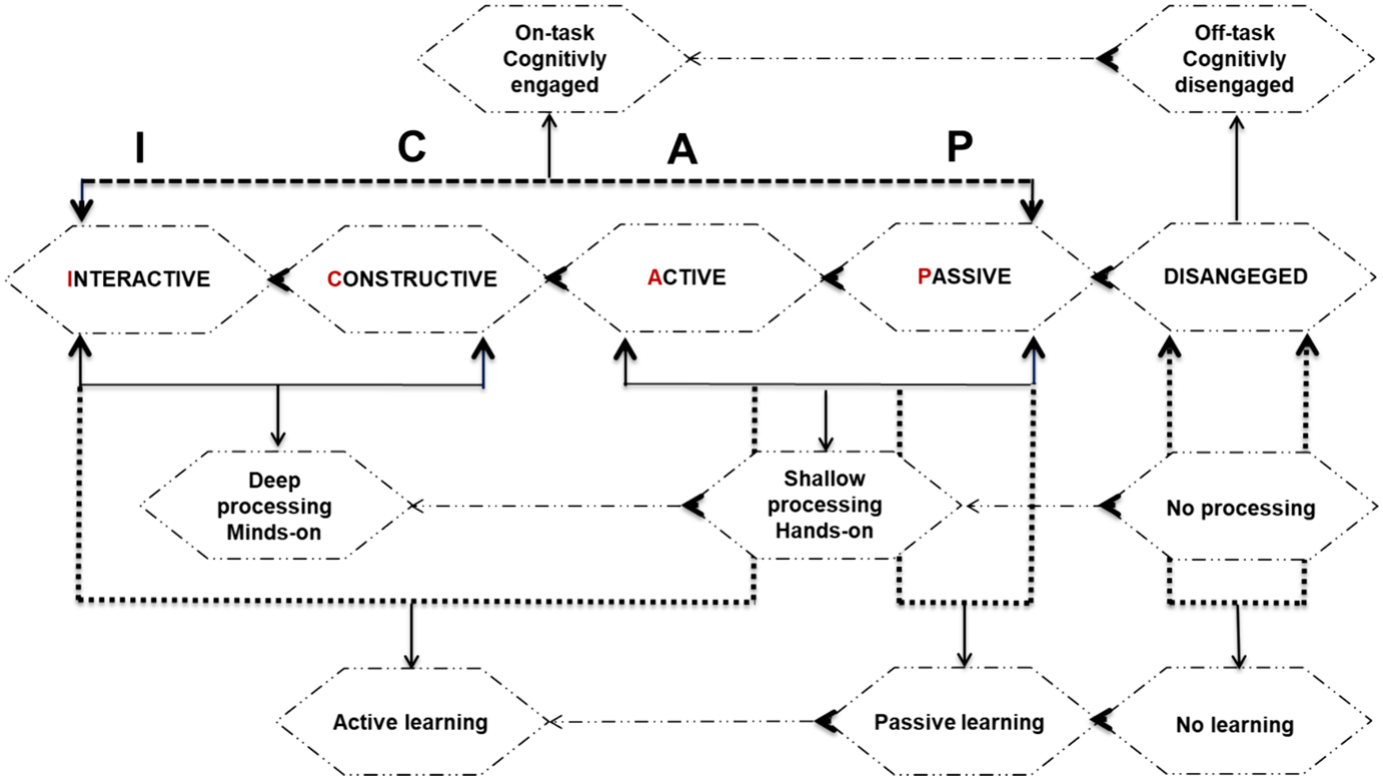
Activities in the learning process—the viewpoint of ICAP theory

ICAP is a theory that defines cognitive engagement or active/passive learning in ways that can promote deeper cognitive processes (Chi, 2009; Chi & Wylie, 2014; Chi et al., 2018). It is a comprehensive theory that determines how students can cognitively engage with teaching material in a concrete and explicit way that can be generalized across student age, content domain, and context. According to this theory, cognitive engagement refers to behaviour, i.e. the process of interacting with instructional teaching material, which is based on specific cognitive processes and implies the investment of cognitive effort in the learning process (Chi et al., 2018). The terminology within this theory describing the learning process and activities can be explained in Fig. 1.

The brief description of Fig. 1 is as follows. The term off-task implies student behaviour manifests itself as cognitive non-engagement (disengaged), while the term on-task manifests itself as cognitive engagement (engaged) within the instructional teaching material. The degree of cognitive engagement ranges from minimum—*passive mode* to maximum—*interactive mode*. The term passive learning implies that the students do nothing with the instructional teaching material, while the term active learning implies that the students do something with the instructional teaching material. Passive learning includes the last ICAP mode *passive* (minimum cognitive engagement), while active learning includes the first three ICAP modes (*active*, *constructive,* and *interactive*). The term shallow processing—hands-on means involving students in more superficial cognitive activities such as *storing*, *activating,* and *linking* (modes *passive* and *active*), while the term deep processing—minds-on means involving students in more profound cognitive activities based on generating and reasoning such as *infer-from-own* and *infer-from-others* (through *constructive* and *interactive* modes).

**Fig. 2:**
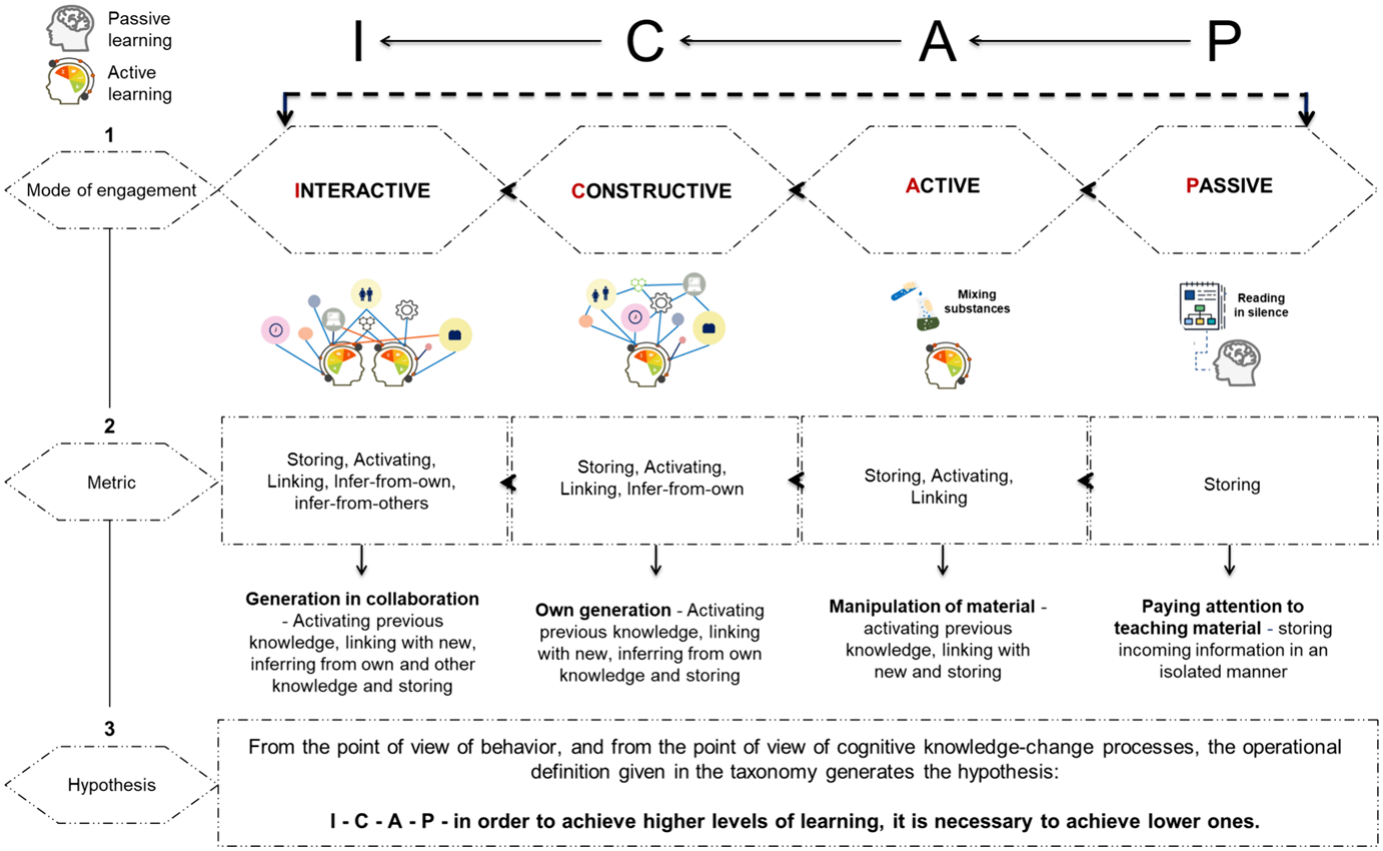
Basic components of ICAP theory

The ICAP theory includes three essential components: 1. a taxonomy of four modes of engagement with an operational definition of each mode; 2. a metric that can define the degree of engagement based on cognitive behaviour, which corresponds to each of those modes of behaviour; 3. as well as a hypothesis that can predict hierarchical levels of student learning, as a function of a particular mode of engagement (Chi et al., 2018; see Fig. 2).

ICAP theory has categorized students' cognitive engagement into four broad modes divided into four sub-scales with clear distinctions concerning overt behaviour and products that students can create or express. Each mode of engagement is characterized by the corresponding underlying cognitive knowledge change processes (see Fig. 2—metric part). We will briefly describe them.

- **Passive-attentive mode (or paying attention mode)**—students are focused on information from the teaching material and receiving it, without overtly doing anything else related to learning (e.g. reading the text silently, watching videos, listening to online lectures…) (Gobert et al., 2015). Role of the teacher—use technology to show and explain content.
- **Active (or manipulating) mode**—students undertake some form of overt action or physical manipulation without providing additional information (e.g. show or gesture what they read or solve, pause or rewind video, rotate an object, mix substances…) (e.g. Chi et al., 2008; Yaron et al., 2010). Role of the teacher—actively use technology with students.
- **Constructive (or generating) mode**—students produce externalized ideas that contain generated information and, in this way, go beyond what is provided in the learning material (they find similarities and differences, self-explain, write in their own words, and ask questions…) (e.g. Schauble et al., 2009; Schwartz et al., 2011). Role of the teacher—monitors student use of technology.
- **Interactive (or collaborating) mode**—students interact with a peer (or within smaller groups) through a dialogue that fulfils two criteria. First—the conversation of both partners must be generative/constructive (ideas go beyond what is present in the learning material). Second—each partner’s contribution deals with or engages the contribution of the other partner so that they mutually generate and co-generate (build on, elaborate, and challenge each other's ideas…) (Antonietti et al., 2023a). Role of the teacher—monitors student use of technology in collaboration.

According to the ICAP assumption, each mode with the corresponding underlying cognitive knowledge change processes has a hierarchical structure in behaviour—so that the lower ones condition the appearance and existence of the higher ones. Levels of student engagement can be observed based on behaviour and the product of their work (Deepika et al., 2021). In this way, ICAP can serve as an aid to teachers in assessing students' cognitive engagement, but also to educators and researchers as a tool for analyzing teacher questions, activities, and comprehensive work.

## The ICAP Technology Scale

The ICAP-TS assesses the quality of TI from the aspect of student cognitive activation (engagement) through the ICAP framework (Chi & Wylie, 2014; Chi et al., 2018). Therefore, it intends to evaluate how teachers integrate technology to encourage and support student cognitive activation, as one of the dimensions of the quality of TI, and does not assess other dimensions of the quality of technology-supported instruction, such as teacher support and classroom management (Runge et al., 2023). Accordingly, we focus precisely on cognitive activation as a dimension of TI quality. Further on in the text, under the term TI quality, we mean the quality seen through this dimension. TI is defined as using technology (hardware and software tools) in an educational context to achieve learning outcomes and support educational goals or as a process that leads to this (Consoli et al., 2023; Fütterer et al., 2024). The quality of TI among other things is reflected in the degree to which it is used to transform and redefine learning activities, but in such a way that they encourage and support cognitive activation (a term defined in the first section of the paper) of students, i.e. initiate in-depth processing of the content (Anđić et al., 2024b; Fütterer et al., 2023a; Maričić & Lavicza, 2024). Cognitive activation is a key driver of a successful learning process in which deeper, invisible aspects of teaching play a decisive role in processing and understanding information and therefore represent a key aspect of teaching quality (Fütterer et al., 2022). It is reflected in the degree to which teacher instruction has the potential to promote students' active cognitive engagement and higher-order thinking (Fütterer et al., 2023a; Praetorius et al., 2018). Therefore, we can define ICAP-TS as an instrument that measures the quality of TI by teachers through the frequency of passive, active, constructive, and interactive learning modes supported by technology, which indicates the level of student cognitive engagement through the underlying cognitive knowledge change process, which correspond to certain types of engagement. In the upcoming section, we will briefly present an overview of previous research constructing and applying different scales that measure the integration and quality of TI in teaching through different theoretical foundations including related work on the ICAP framework.

## Literature review

As mentioned earlier, previous research on this topic used either simplified or quite complex (difficult to interpret) techno-centric scales for evaluating TI in teaching. Their aim is mainly focused on embracing integration, determining the type of digital tools and software that teachers and students use in the classroom (e.g. Gomez et al., 2022; Scherer et al., 2020), and how often they use them in the learning process (e.g. Fraillon et al., 2020). The examination of these TI variables was carried out through teachers and student’s perceptions, attitudes, beliefs, and knowledge (Agyei & Voogt, 2011; Farjon et al., 2019; Mourlam et al., 2019; Newland et al., 2018; Wilson, 2021). Although these scales can be used to measure and analyze the frequency and familiarity of technology use, they cannot be taken as a direct indicator of the quality of technology use in teaching and learning (Antonietti et al., 2023a). The reason for this is that such scales focus on specific digital tools, and this focus is unlikely to reveal information about how technology is integrated to support learning activities and trigger cognitive activation. In addition to this, the indicator of technology use frequency is insufficient to understand the pedagogy underlying TI, making it difficult to assess the quality of TI. In a preceding meta-analysis on this topic by Consoli et al. (2023) which reviewed 36 studies and 35 TI measurement instruments found that these scales focused on using technology to: improve students' cognitive engagement, promote collaboration between students, and enable students to research the network. Therefore, this systematic review showed that instruments measuring pedagogical and other aspects of TI quality had been used for several years (e.g. Blau & Shamir-Inbal, 2017; Thannimalai & Raman, 2018). However, the problem is that these instruments are based on specific programs (or curricula), so they cannot be generalized. Also, these scales do not contain conceptual or theoretical reflections on the quality of TI. For this reason, they were probably not even considered in the current debate on the quality and quantity of TI (Men & Noordin, 2019). Given that this meta-analysis was published in January of 2023, in the meantime, several studies were published whose instruments also pointed to the pedagogical dimensions, and quality of TI and they are grounded in theoretical frameworks (Antonietti et al., 2023a, 2023b; Backfisch et al., 2021; Fütterer et al., 2022, 2023a, 2023b; Juuti et al., 2022; Ninković et al., 2023; Sailer et al., 2021a; Schmitz et al., 2023, 2024; Wekerle & Kollar, 2022). We will discuss these dimensions in the following paragraphs.

In the study by Fütterer et al. (2022) perceived cognitive activation during the integration of technology, as a dimension of teaching quality, was evaluated with the Genetic-Socratic Procedure scale (Rakoczy et al., 2005), which measures the extent to which students perceive the tasks given in class as an incentive for independent thinking and problem-solving. This study approached the measurement of the quality of TI through the concepts of perceptions and attitudes about TI that have previously been shown to be related to achievement and not commensurate with actual behaviours (e.g. Lee et al., 2019). Considering that, it was established that more direct ways are needed to measure the quality of TI, which, in addition to the affective component, are also connected to the experiential one. In the study by Juuti et al. (2022), the perceived quality of TI by students was evaluated through Martin Heidegger's (1962) general analysis of the functional use of tools and appliances, within which two activities are distinguished—readiness-to-hand (technology is seen as a piece of equipment that serves a specific purpose) and presence-at-hand (technology is viewed as an object with specific properties). The quality of TI was assessed through the students’ task to express their experience in working with technology for each of the 10 items within both activities. This scale is focused only on two different types of activities, which do not go into deeper cognitive processes, and student perceptions, which do not provide much information about the quality of teachers’ technology integration. In addition to these theoretical models, technology integration is also measured through the most prominent model for this type of assessment in educational research—the "will, skill, tool" (WST) model (see Fütterer et al., 2023b; Schmitz et al., 2024).

In addition to these, several studies have used the ICAP framework as a conceptual model for developing TI quality measurement instruments (Deepika et al., 2021; Fütterer et al., 2023a; Sailer et al., 2021a, 2021b; Stegmann, 2020; Wekerle & Kollar, 2022; Wekerle et al., 2022). On the one hand, these instruments are either aimed at classifying and differentiating technology-supported activities that students have shown through their cognitive engagement (Deepika et al., 2021; Stegmann, 2020) or students' attitudes about the frequency of TI within each ICAP sub-scale to assess their cognitive engagement (Fütterer et al., 2023a; Wekerle & Kollar, 2022; Wekerle et al., 2022), which focuses on the student's perspective and does not provide insight into the quality of TI by teachers. On the other hand, these instruments contain only one or few items within each sub-scale of engagement (Sailer et al., 2021a), which makes it difficult to measure the frequency of TI. At the beginning of 2023, based on the ICAP pedagogical principles Antonietti et al. (2023a) developed a theoretically-based instrument with a scale to effectively measure how teachers integrate technology in various teaching and learning activities—the so-called ICAP-TS. In addition to constructing the instrument, the authors tested it on a sample of 1059 teachers from Switzerland (we will focus on this in more detail in the discussion). In May 2023, the first paper that tests this instrument—ICAP-TS by Ninković et al. (2023) was published. Within it, teachers were supposed to indicate which types of activities/modes they most often use technology. In addition to the cognitive activation, other dimensions of TI quality were also measured—the correlation between innovative school climate, the principal's support, and the use of technology in various ICAP activities (the results of this paper we will mention in the discussion). This issue is further elaborated in the work of Schmitz et al. (2023) within which the variables that mediate the relationship between transformational leadership and technology integration were identified. Testing of the scale continued also through the work of Antonietti et al. (2023b) in which the relationship between technology-related professional development and the quality of technology integration was investigated.

### The present study

Recommendations of previous research led us to the aim of our study. In addition to that, the originality of this paper is reflected in the provision of a completely new contribution to science within this issue. We will explain these two facts briefly in the text below.

*Recommendations from previous research:* Given that the ICAP-TS instrument was recently developed, its metric characteristics were not tested across different languages and on diverse samples of teachers. In recommendations for future research, Antonietti et al. (2023a) stated that it was necessary to test the ICAP-TS among teachers from different educational contexts to evaluate whether or not this scale could also be used in primary and higher education research. In a recent study on this issue by Ninković et al. (2023), ICAP-TS showed good model fit. However, the authors observed a high level of correlation between constructive and interactive use of technology in teaching indicating that these two sub-scales are not separated which may threaten the discriminant validity of the scale. For these reasons, verifying this fact on a diverse sample is recommended. Also, within previous research, the quality of TI of teachers from one country (Antonietti et al., 2023a; Ninković et al., 2023) and a small number of schools were examined (Ninković et al., 2023). Following the recommendations for future research, it was suggested to go further, because insights into how a larger number of teachers integrate technology into different teaching and learning activities are needed to obtain reliable results, not reducing the statistical power of the tests used, compare them and draw suitable recommendations for practice, theories, and future studies.

Based on these recommendations, we have formulated the following parts of our aim: Firstly, we aspired to verify the scale's construct validity and reliability on a large sample of teachers across seven European countries. Secondly, we aimed to evaluate the quality of TI on this heterogeneous sample with the ICAP-TS to explore for which learning activities teachers use technologies, within which ICAP learning modes, and how often to encourage and support cognitive activation of their students.

**Fig. 3:**
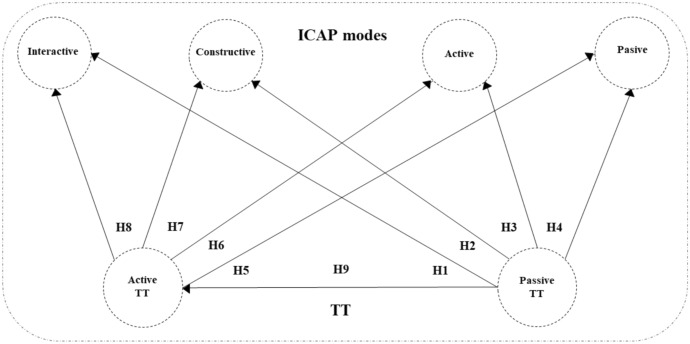
The research model

*The originality of our study*: Given that the scale consists of 12 items that measure the integration of technology through various learning activities (interactive, constructive, active, and passive), a part of another 12 items was added to the original scale, which refers to the integration of concrete—the most commonly used technologies in teaching practice (Antonietti et al., 2023a; Fütterer et al., 2023a). In this way, two main constructs of the scale were created—the ICAP construct and the TT construct. This organization of the scale gave us a chance to explore the interplay i.e. the relationships between these constructs, which can provide us with an insight into which TTs predict which ICAP modes in teaching practice, which is of great importance for practitioners. This issue has not been investigated in the literature so far, and with our study, we want to fill that research gap. The proposed research model is shown in Fig. 3.

**Fig. 4:**
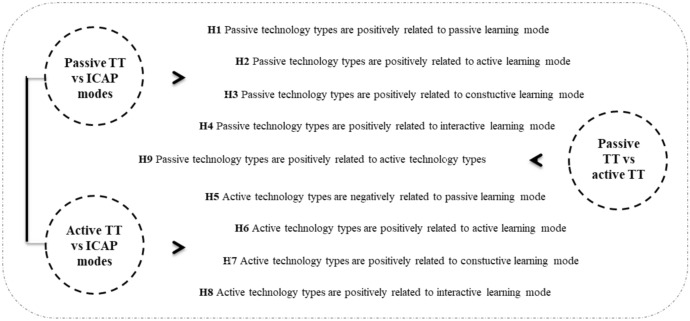
Research hypotheses

To establish these relationships (correlations), we had to test the hypotheses shown in Fig. 4.

Based on the explanation of the originality of this study, the last part of our aim emerged. Thirdly, we strived to examine relationships between different TT and TI in ICAP learning modes.

## Methodology

### Research design

**Fig. 5:**
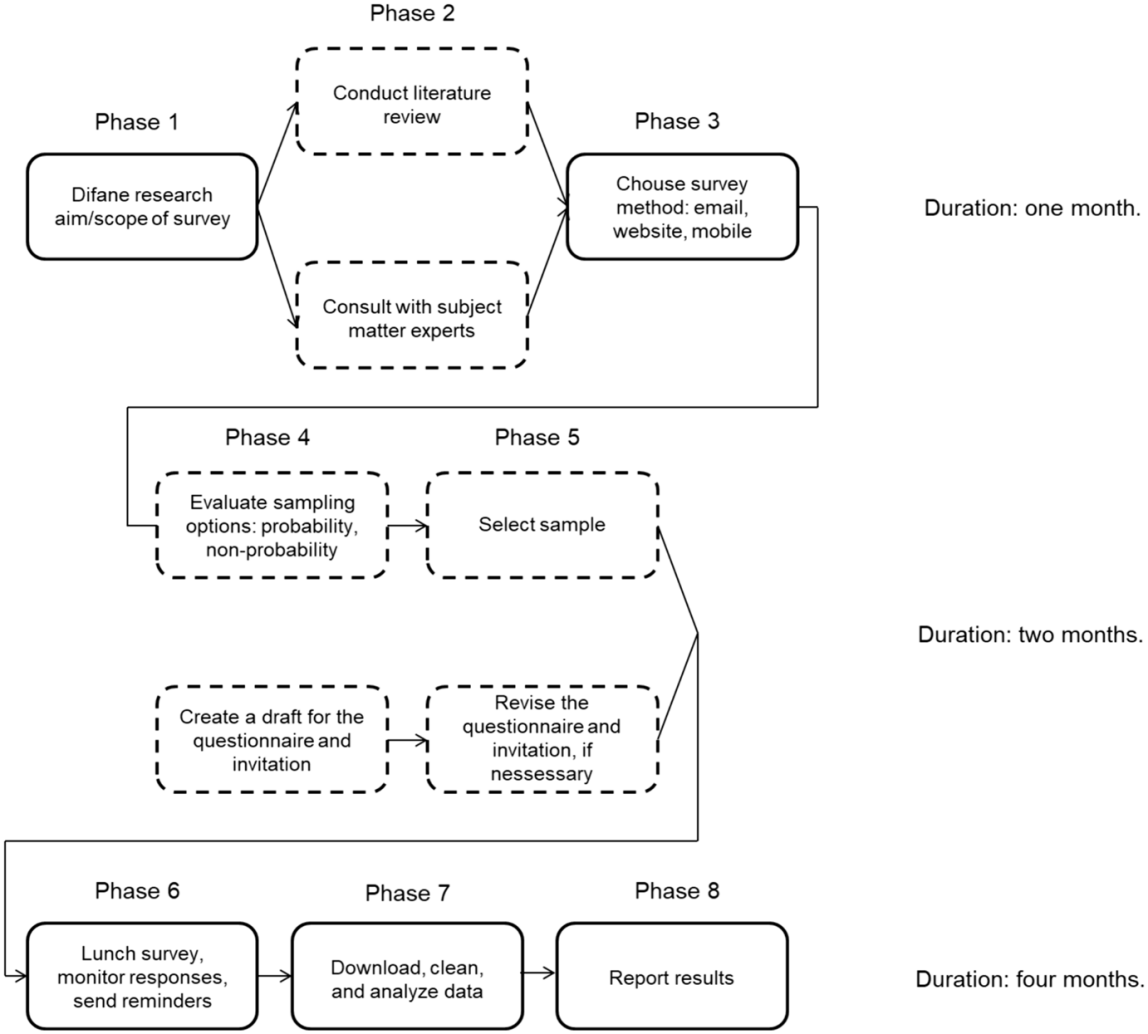
Research phases

The research was carried out using a survey research design. This type of design uses the respondents' statements about their perceptions—opinions, attitudes, beliefs, and behavior—as the main source of data (Knežević-Florić & Knežević, 2012; Vomberg & Klarmann, 2022). For our research, an analytical (explanatory) survey was selected, which follows the logic of experimental research, checks the set hypotheses, and establishes cause-and-effect relationships (Knežević-Florić & Knežević, 2012). The surveying technique with the instrument questionnaire was applied because, in this way, a large amount of data can be collected in a short time with relatively low costs. The phases of the research are shown in Fig. 5.

Within the 1st phase, the aim of the research was defined. In the 2nd phase, we reviewed the relevant literature within the scope of the survey. Through this phase, we got acquainted with the opinions of subject matter experts, and based on that we developed a three-fold aim (a detailed description of 1st and 2nd phases is given in the Theoretical background). In the 3rd phase, we chose the e-mail survey method. This method was chosen for the following reasons: it ensures the anonymity of respondents, it is flexible, it is fast when collecting answers, the cost of implementation is very low, there is no interviewer bias, and does not tend to give socially desirable answers. In the 4th phase, we opted for a non-probability sample, using the convenience sampling method, because in this way we could access all the respondents available to the researcher. Then, we created a draft version of the questionnaire (which is a translated and adapted version of the Antonietti et al., 2023a questionnaire) and an invitation letter. This was followed by the translation and standardization of conceptual equivalences on these documents, which we adapted to the languages of different countries. In the 5th phase, teachers of primary and lower-secondary schools were selected for the sample and the final version of the questionnaire and invitation letter was created. In the 6th phase, a questionnaire with an invitation letter was sent to respondents via e-mail. This phase included monitoring the collection of responses, as well as sending friendly reminders (with an indication that respondents who had not previously filled in the questionnaire should complete it). In the 7th phase, we downloaded the respondents' answers. This was followed by their cleaning, arrangement, and detailed analysis. As a result of this, in the 8th phase, we obtained results that we explained in the form of a final report. We will describe the 4th, 5th, 6th, 7th, and 8th phase in more detail in the text below.

### Sampling and data collection

A non-probability sampling method, specifically convenience sampling, was employed. This method was chosen because it allowed us to include respondents who were readily accessible to the researchers. Convenience sampling is practical and cost-effective, especially when the study aims to gather data from a specific, accessible population. Given that the aim of the research is focused on examining the integration of technology by teachers, by the recommendations from previous research, we decided on a sample that this issue has not yet covered. The study involved primary and lower secondary school teachers from seven European countries: Austria, Czech Republic, Croatia, Montenegro, Republic of Serbia, Slovenia, and Turkey. These countries were selected based on existing connections with colleagues, which facilitated access to a large number of teachers. Nevertheless, after reporting on the initial results and refining our approaches data collection will be extended to other countries to enhance our work further. The data collection involved the active participation of at least one experienced bilingual researcher who possesses in-depth knowledge about the educational system of the participating country and its unique characteristics.

A similar data collection procedure was used in all countries with slight alternations to local needs. Before developing our research instrument, permission was sought from the authors (Antonietti et al., 2023a) to use and adapt the scale to the native languages of teachers from all participating countries. In the beginning, a research questionnaire was developed in the English language. Then the questionnaire was translated into the native language of teachers from all countries involved in the research. This translation was done by the author/researcher from each country with one colleague—an expert in the English language and native language. During this process, linguistic and conceptual cross-cultural differences in the formation of questionnaires (Osborn, 2004) and their interpretation/translation were carefully considered resulting in several revision cycles. Adaptations were carried out simultaneously for all countries with the agreement and discussion of the team members (authors of the paper/researchers) about these meanings and conceptual equivalences. Given that all researchers from these seven countries are bilingual (in addition to their native language, they speak English) and that they had the help of an expert in the English and native language, we avoided the need for interpreters (Osborn, 2004). After translation, the instrument was checked and assessed for clarity by trained educational research experts from each country. Based on the opinion of experts, the clarity of the instrument has been improved. After that, the validity of the questionnaire was checked by 10 teachers with longer working experience from each of the countries. These teachers were asked to give their opinion on the meaningfulness, relevance and clarity of the questionnaire. Based on teachers' feedback, the final questionnaire was created and this was followed by the actual data collection. During this procedure, feedback from experts and teachers was of a linguistic, grammatical and syntactic nature ensuring the validity of the questionnaire. Thus, the final version of this scale did not differ from the one presented in the paper of Antonietti et al. (2023a).

Data collection was conducted using an online google questionnaire. As explained earlier, we had access to teacher lists from our connections in countries and sent out invitations to participants often following methods inspired by snowball sampling (Cohen et al., 2017). The data collection was carried out during a three-month period. An email explaining the purpose of the data collection and adding a link to the questionnaire was sent to school leaders in all countries. Two weeks after the first email, a friendly reminder was also sent. Fifteen days after the reminder email, the questionnaire was closed and data collection was completed. A total of 2550 teachers responded to the questionnaire voluntarily. After excluding outliers and missing data, 2277 teachers were included in the final analysis. The demographics of the participants are shown in Table 1.

**Table 1:** Demographic characteristics (*N* = 2550)

Variable	Frequency	%
*Gender*		
Female	2029	79.6
Male	476	18.7
Missing	45	1.8
*Age*		
20–30	317	12.4
31–40	569	22.3
41–50	809	31.7
50 +	855	33.5
*Experience*		
< = 5	389	15.3
6–10	274	10.7
11–15	306	12.0
16–20	324	12.7
20 +	1257	49.3
*Profession*		
Subject Teacher	1480	58.0
Class Teacher	1049	41.1
Missing	21	.8
*Country*		
Austria	355	13.9
Czech Republic	419	16.4
Croatia	229	9.0
Montenegro	227	8.9
Republic of Serbia	887	34.8
Slovenia	222	8.7
Turkey	211	8.3

The basic condition for the inclusion of teachers in the research was their voluntary consent. Confidentiality of collected data and preservation of anonymity are guaranteed to all participants. We will talk about this in more detail in the description of the instrument.

### Instrument

The questionnaire was designed to include two sections—the introductory (informative) section and the central (scale) section. In the introductory part of the questionnaire, the ICAP framework with the meaning of all four different sub-scales is explained. Below these explanations, it is indicated that participation in the research is voluntary, that each respondent is guaranteed anonymity (without the possibility of revealing their identity) and that in this way we collect unrepresentative but valuable data for our exploratory correlational study. The central part of the questionnaire is organized into three parts: the first part—is demographic characteristics of teachers (age, gender, years of professional experience, subject taught by teachers); the second part—items used as measures for the ICAP-TS (ICAP construct), and the third part—list of 12 most commonly used technology types in teaching (TT construct). Further, we will briefly describe the second part of the questionnaire which consists of two constructs.

*The ICAP construct*—ICAP-TS consists of 12 items evenly distributed across all four sub-scales of different ICAP learning modes. Within each sub-scale, three items measure how often teachers integrate technology and within which learning activities to encourage and support the cognitive activation of their students. The passive learning sub-scale describes activities in which teachers use technology to show and explain instructional content, and students are in a receptive learning position (*storing*). The active sub-scale describes teachers' and students' active use of technology in which students apply previously acquired knowledge (*storing, activating* and *linking*). The constructive sub-scale describes activities in which students use technology to acquire new knowledge (*storing, activating, linking*, and *inferring-from-own*). The interactive sub-scale describes cooperative learning activities, which are the context of acquiring new knowledge through student cooperation and collaboration (*storing, activating, linking*, *inferring-from-own* and *inferring-from-others*)*.*

*TT construct*—A part of another 12 items related to the integration of different—most commonly used technologies in education was added to the ICAP scale: learning programs or subject-specific software, online exams and quizzes, word processing, spreadsheet or calculations, games, internet learning platforms, drawing and image editing, video recording/video editing, research on the internet, online communication during class, presentation/publication of student work, and learning management system.

For each item on the questionnaire, teachers were asked to indicate on a five-point Likert scale (1—almost never to 5—almost every lesson) how often they use technology within each of the activities and how often they integrate certain technology types.

### Data analysis

After downloading, cleaning, and arranging the data, their detailed analysis followed. Since the kurtosis and skewness values were between + 1.5 and -1.5 (data are normally distributed), we used parametric techniques (Tabachnick et al., 2013). We used various statistical techniques to analyse the data collected from the survey. Descriptive statistics were employed to summarize the basic features of the dataset, providing an overview of the sample and measures. This included calculating frequencies, means, and standard deviations to understand the distribution of responses. Confirmatory Factor Analysis was conducted using SmartPLS 4 to assess the construct validity and reliability of the ICAP-TS across different countries. This technique helps verify the factor structure of the measurement instrument, ensuring that it accurately reflects the constructs it is intended to measure. Additionally, Exploratory Factor Analysis was performed with SPSS 25 to identify the underlying components of the technology types (TT) used by teachers. Principal component analysis was used as part of EFA to reduce data dimensions and identify key factors. To examine differences in technology integration patterns across countries, Analysis of Variance (ANOVA) was applied. Due to non-homogeneous variances, Welch’s t-test was used instead of the traditional F statistic, ensuring robustness in the presence of heteroscedasticity. Partial Least Squares Structural Equation Modeling (PLS-SEM) was utilized with SmartPLS 4 to explore the relationships between different TT and ICAP learning modes. PLS-SEM is suitable for complex models and helps in understanding the predictive relationships among variables.

We adopted the steps proposed by Hair et al., (2021a, 2021b) to evaluate reliability and validity:

- *Indicator reliability*: Factor loadings higher than 0.70 indicate a relatively high relationship between the item and the factor (Hulland, 1999). Indicators with very low loadings (below 0.40) should permanently be eliminated from the measurement model (Hair et al., 2022).
- *Internal consistency reliability*: Composite Reliability (CR) was used to determine the reliability of each item and construct in the model. Low convergent validity indicates that the items contain information from additional factors besides the related factor. Cronbach's Alpha is used to determine internal consistency. While Cronbach's Alpha tends to underestimate the reliability of latent variable scores, CR (rho_c) tends to overestimate their reliability. In contrast, the novel reliability coefficient CR (rho_a) accurately reflects the actual reliability of construct scores (Dijkstra & Henseler, 2015). It is recommended that the internal consistency reliability values should be above 0.70 for Cronbach's Alpha and CR (rho_c) (Hair et al., 1998; Thompson et al., 1995).
- *Convergent validity*: This analysis was assessed using the Average Variance Extracted (AVE) (Bagozzi & Yi, 1988; Hair et al., 2010). AVE corresponds to the commonalities of a construct. A minimum acceptable AVE value is 0.50, which means an AVE of 0.50 or greater signifies that the construct accounts for 50% or more of the variances among the indicators that constitute the construct (Hair et al., 2021a, 2021b).
- *Discriminant validity*: It was assessed using the Heterotrait-Monotrait ratio of correlations (HTMT). When the HTMT value is less than 0.90, it indicates discriminant validity between the two variables (Henseler et al., 2015).

Following the aforementioned data analyses guidelines, in the next section, we will present results (our final report) and then discuss them in the following three subsections each of which follows a part of the stated three-fold aim: 1st part, 2nd part and 3rd part.

## Results

### ICAP-TS construct validity and reliability

**Fig. 6:**
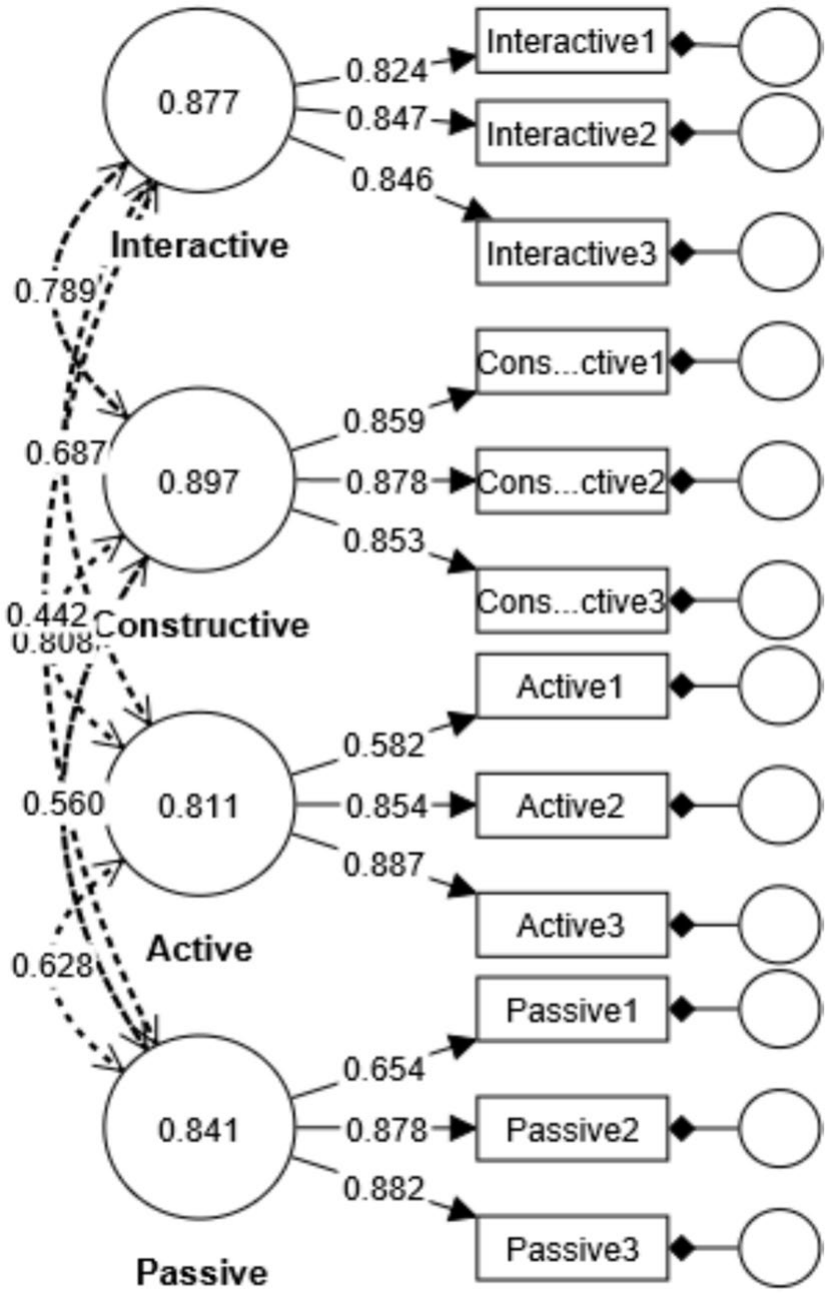
Measurement model of ICAP scale

*The 1st part*: results of Confirmatory factor analysis were examined in terms of various fit indices, which are regularly used in the evaluation of the model fit. Fit indices: comparative fit index—CFI, and goodness of fit index—GFI range from 0 to 1. Values between 0.90 and 0.95 are acceptable, while above 0.95 indicates a good fit (Bentler, 1990; Hu & Bentler, 1999; Marsh et al., 2004). Based on these fit indices, the ICAP scale for Montenegro and Croatia fits the four-factor model poorly (Appendix Table 4). The ICAP scale for Slovenia fits the model only slightly. The ICAP scale for Austria, Czech Republic, Republic of Serbia, and Turkey samples fit the model well. The fit indices also show that the ICAP scale fits the overall sample well (χ^2^ = 623.178, *df* = 48.00, *p* = 0.000; RMSEA = 0.073, SRMR = 0.042, GFI = 0.957, CFI = 0.969) (Fig. 6).

The correlation between the item and the factor is measured by factor loading; a factor loading greater than 0.30 usually indicates a moderate correlation between the item and the factor (Shevlin & Miles, 1998). Factor load ranges for Austria, Czech Republic, and Montenegro are larger than for other countries. AVE values were calculated above 0.50 except for Montenegro and Austria for interactive sub-scale and Czech Republic for active sub-scale. Low CR values (below 0.70) were observed only for Montenegro for interactive sub-scale and Czech Republic for active sub-scale (see Appendix Table 5). Cronbach's Alpha values for ICAP sub-scales by countries were calculated higher than 0.70 except Czech Republic for the active sub-scale. The HTMT values for the constructive sub-scale were calculated above 0.90 but below 1.00 for all countries except Czech Republic and Croatia. This shows that discriminant validity by country is critical for constructive sub-scale. Also, it was observed that discriminant validity of the passive sub-scale has critical values for Montenegro. The correlation between interactive and constructive sub-scale is comparatively high.

### TT, ICAP learning/engaging modes, and its frequency

*The 2nd part*: We will organize this subsection in three units—TT, differences in ICAP constructs by country and differences in TT by country.

#### TT

To understand the various components contributing to teachers’ use of different TT, Principal component analysis was conducted. Before conducting principal component analysis, descriptive statistics were used to test the data for fit (Appendix Table 6). The Kaiser–Meyer–Olkin test (= 0.912) and Bartlett's test for sphericity (*p* < 0.000) show that the correlation matrix of the data is suitable for factor extraction. Principal component analysis revealed two components with eigenvalues greater than 1, which explained a cumulative variance of 57.15%. The factor loads are above 0.60 (Appendix Table 7). The first component explains 29.05% of the total variance, and the second explains 28.01%. Cronbach's Alpha for the first component was calculated as 0.85, and for the second component 0.84. Component-1 is labelled as *passive* TT and Component-2 as *active* TT. These labels are used in the following analyses.

#### Differences in ICAP constructs by country

Descriptive statistics for ICAP-TS by country are presented in Appendix Table 8. Croatia and Turkey have the highest mean values for all four sub-scales. Among the learning modes, Austrian teachers have the highest mean value for the interactive learning mode, while teachers from Czech Republic, Croatia, Republic of Serbia, Slovenia, and Turkey have the highest mean value for the passive mode. Austrian teachers have the lowest mean value for constructive learning mode, while teachers from Czech Republic, Croatia, Republic of Serbia, and Slovenia have the lowest mean value for interactive learning mode. Turkey teachers have the lowest mean value for active learning mode. Montenegro has almost similar mean values for all sub-scales. ANOVA analysis was performed to examine whether there was a significant difference between countries for the ICAP-TS means. Since the variances are not homogeneous, the results were interpreted with Welch's t-test instead of the F statistic (Kohr & Games, 1974). The results showed that there are significant differences between countries for ICAP-TS (interactive *Welch’s F* (6, 2270.00) = 18.21, *p* = 0.000, constructive *Welch’s F* (6, 2270.00) = 39.15, *p* = 0.000, active *Welch’s F* (6, 2270.00) = 19.13, *p* = 0.000 and passive *Welch’s F* (6, 2270.00) = 64.32, *p* = 0.000). Tamhane's T2 test was preferred to examine between which groups there is a difference. These results are presented in Appendix Table 9. Croatia, Austria, and Turkey lead in TI into interactive learning mode. Croatia, Turkey, and Republic of Serbia lead in TI into constructive learning mode. Croatia, Czech Republic, and Turkey lead in TI into active learning mode. Turkey, Croatia, and Slovenia lead in TI into passive learning mode.

#### Differences in TT by country

Descriptive statistics for TT by country are presented in Appendix Table 10. Austria and Montenegro have the highest mean values for active TT, while teachers from Turkey, Croatia, Republic of Serbia, Czech Republic, and Slovenia have the highest mean values for passive TT. ANOVA analysis was performed to examine whether there was a significant difference between countries for different TT. Since the variances are not homogeneous the results were interpreted with Welch's t-test. The results showed that there is a significant difference between countries for TT (passive *Welch’s F* (6, 2219.00) = 74.40, *p* = 0.000, active *Welch’s F* (6, 2227.00) = 71.63, *p* = 0.000). Tamhane's T2 test was preferred to examine between which groups there is a difference (Appendix Table 11). There is a significant difference between Austria, Turkey and the other countries for passive TT (except Turkey and Croatia) and between Austria and the other countries for active TT (except Austria and Turkey). Turkey, Croatia, and Republic of Serbia lead in passive TT while Austria and Turkey lead in active TT.

### Relationships between TT and TI in ICAP learning/engaging modes

#### Measurement model

*The 3rd part*: The relationship of the constructs in the measurement model to a common variable was examined through the full collinearity method used for common factor bias (Kock, 2015). Calculated variance inflation factor values for the constructs show that common method bias does not jeopardize the findings. Factor loadings were found above the recommended value, except EdTech11 (0.689). Internal consistency reliability and AVE values are shown in Table 2.

**Table 2:** AVE and internal consistency reliability values of the model

Constructs	Item	Cronbach's Alpha	CR (rho_a)	CR (rho_c)	AVE
Interactive	Interactive	.877	.878	.924	.802
	Interactive				
	Interactive				
Constructive	Constructive	.897	.898	.936	.830
	Constructive				
	Constructive				
Active	Active1	.811	.856	.888	.727
	Active1				
	Active1				
Passive	Passive	.841	.843	.905	.760
	Passive				
	Passive				
Passive TT	EdTech1	.850	.853	.889	.572
	EdTech2				
	EdTech3				
	EdTech5				
	EdTech6				
	EdTech9				
Active TT	EdTech4	.834	.835	.879	.548
	EdTech7				
	EdTech8				
	EdTech10				
	EdTech11				
	EdTech12				

According to Table 2, the Cronbach's Alpha values range between 0.811 and 0.897. CR values are between 0.835 and 0.936. CR (rho_a) values are above 0.80 (Dijkstra & Henseler, 2015). AVE values were calculated above 0.50 (Hair et al., 2019). All HTMT values were calculated below 0.85 (Appendix Table 12).

#### Structural model

Variance inflation factor values for each indicator were calculated and found below 3. Results (Table 3) show that all hypotheses were supported (SRMR = 0.045, χ^2^ = 3085.932, NFI = 0.905). Passive TT is positively related to TI in passive learning mode (β = 0.624, *p* < 0.000), active learning mode (β = 0.468, *p* < 0.000), constructive learning mode (β = 0.437, *p* < 0.000), and interactive learning mode (β = 0.184, *p* < 0.000) (H1, H2, H3, and H4). Active TT are negatively related to TI into passive learning mode (β = -0.054, *p* < 0.000) but also positively related to TI into active learning mode (β = 0.123, *p* < 0.000), constructive learning mode (β = 0.186, *p* < 0.000), and interactive learning mode (β = 0.442, *p* < 0.000) (H5, H6, H7, and H8). Passive TT are also positively related to active TT (β = 0.638, *p* < 0.000) (H9).

**Table 3:** Hypothesis testing results (direct effects)

Hypothesis (H)	Path	β	*SD*	*t*	*p*	If H supported	*f*^*2*^
H1	Passive TT—> Passive	.624	.020	31.431	.000	Yes	.355
H2	Passive TT—> Active	.468	.024	19.815	.000	Yes	.188
H3	Passive TT—> Constructive	.437	.023	19.308	.000	Yes	.169
H4	Passive TT—> Interactive	.184	.023	8.086	.000	Yes	.030
H5	Active TT—> Passive	− 0.054	.020	2.663	.008	Yes	.003
H6	Active TT—> Active	.123	.025	4.904	.000	Yes	.013
H7	Active TT—> Constructive	.186	.023	8.255	.000	Yes	.031
H8	Active TT—> Interactive	.442	.022	20.559	.000	Yes	.174
H9	Passive TT—> Active TT	.638	.013	48.276	.000	Yes	.687

Effect size (*f*^*2*^)—small (> = 0.02), medium (> = 0.15), or large (> = 0.35)) for each hypothesis: H5 (*f*^*2*^ = .003) and H6 (*f*^*2*^ = .013) small effect size; H2 (*f*^*2*^ = .188), H3 (*f*^*2*^ = .169), H4 (*f*^*2*^ = .030), H7 (*f*^*2*^ = .031), and H8 (*f*^*2*^ = .174) medium effect size, and H1 (*f*^*2*^ = .355) and H9 (*f*^*2*^ = .687) large effect size

**Fig. 7:**
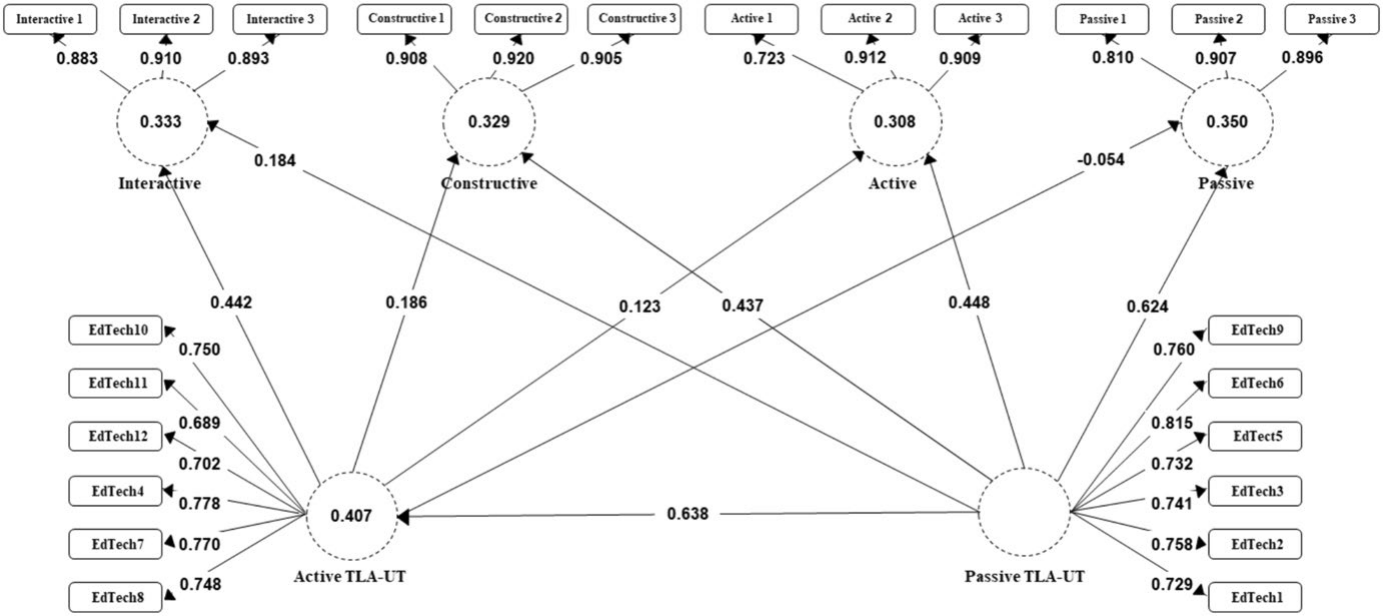
Structural model

Figure 7 represents the results obtained from the hypotheses presented in the structural model.

The results of *R*^*2*^ and *Q*^*2*^ values indicate the predictive relevance in the model. The in-sample predictive power of the model for the constructs is medium (passive *R*^*2*^ = 0.350, active *R*^*2*^ = 0.308, constructive *R*^*2*^ = 0.329, interactive *R*^*2*^ = 0.333). The variances of the constructs vary from 32.9% to 40.7%. The out-of-sample predictive power of the model for the interactive endogenous construct is moderate (*Q*^*2*^ = 0.216), while for constructive (*Q*^*2*^ = 0.308), active (*Q*^*2*^ = 0.298), and passive (*Q*^*2*^ = 0.347) constructs is large.

## Discussion and conclusions

### ICAP-TS construct validity and reliability

*The 1st part*: results of our research showed that the ICAP-TS model fits the overall sample well i.e. is well aligned. However, when this model is considered within each country separately, it can be noticed that for Montenegro and Croatia is critical, for Slovenia is slightly acceptable, while for Austria, Czech Republic, Republic of Serbia, and Turkey is good. This leads us to the need for revision and refinement of the adaptation of the scale for Montenegro and Croatia. Weaker convergent validity was observed for Austria, Montenegro, and Czech Republic compared to the other countries. For Austria and Montenegro, convergent validity is critical for the interactive sub-scale, while for Czech Republic, it is critical for the active sub-scale. Also, this imposes the need to revise the mentioned sub-scales within the model for these countries. The reliability of the scale proved to be satisfactory for all countries. Slightly lower reliability was observed for Czech Republic (active sub-scale) and Montenegro (interactive sub-scale) but only for the one sub-scale within the model, which does not impair the reliability of the whole scale for these countries. Although ICAP-TS is reliable for the countries where the research is conducted, it must be reviewed for validity. Furthermore, discriminant validity was good at the general level. These results are consistent with previous research where ICAP-TS was validated in an educational context in Switzerland (Antonietti et al., 2023a) and Republic of Serbia (Ninković et al., 2023). However, at the sub-scale level the discriminant validity for the constructive sub-scale in all countries except Czech Republic and Croatia, as well as the passive sub-scale for Montenegro is critical. In addition, the differences between: constructive-active sub-scale for Austria; constructive-active, passive-active, passive–constructive, passive-interactive sub-scale for Montenegro; and interactive-constructive sub-scale for Republic of Serbia, Slovenia, and Turkey are not noticeable (due to high correlation), which compromises the discriminant validity of the scale for these countries. A similar observation was also made in the research (Ninković et al., 2023), showing that the interactive and constructive sub-scales for Republic of Serbia are highly correlated. These may be due to the semantic translation of the items into different languages.

### TT, ICAP learning/engaging modes, and its frequency

*The 2nd part*: This part of the discussion is organized into three subsections, which follow the results of the research.

#### TT

Principal component analysis extracted two basic components that contribute to the variation of TT. Within the first component, labeled as *passive* TT the following six technologies where classified: internet learning platforms, games, online tests, and quizzes, specific-subject learning software, online research software, and word-processing software. Within the second component, labeled as *active* TT the following six technologies where classified: online communication software, spreadsheet and calculation software, student work presentation/publication software, learning management system, and video recording and editing software. These components were used in the further interpretation of the results.

#### Differences in ICAP-TS constructs by country

By analysing descriptive statistics, it can be seen that teachers from the Austrian context most often integrate technology so that students have an interactive role (then passive), teachers from the Czech Republic, Croatian, Republic of Serbia, Slovenian, and Turkish contexts most often passively, while teachers from the Montenegrin context most often integrate technology so that students have a constructive (then passive) role in teaching, On a general level—through the analysis of results from all countries, it can be observed that teachers most often passively integrate technology. These results are in line with the results of previous research, in which it was shown that teachers from Switzerland (Antonietti et al., 2023a), Republic of Serbia (Ninković et al., 2023) and Germany (Sailer et al., 2021a, 2021b) most often integrate technology so that students have a passive role in teaching. In the literature, there are two generally accepted reasons why teachers most often integrate technology in a passive way. The first—technological devices and software in the educational environment mainly deal with the use of technologies focused on at teachers and their lectures. Second—significantly less time, resources and competences are required in the usage of technologies for passive learning activities, while active learning activities require more time, resources, as well as high skills in using more sophisticated tools (Antonietti et al., 2023a; Cattaneo et al., 2022; Lohr et al., 2021). Further analysis revealed that teachers from the Austrian context integrate technology the least in a constructive way, teachers from Czech Republic, Croatian, Montenegrin, Republic of Serbia, and Slovenian contexts in an interactive way, while teachers from Turkey integrate technology the least in an active (then interactive) way. On a general level—through the analysis of results from all countries, it can be observed that teachers integrate technology the least in an interactive way, which requires the most time and competence from them, and the greatest cognitive engagement from the students. These results are consistent with the results of previous research (Antonietti et al., 2023a; Fütterer et al., 2023a; Ninković et al., 2023; Wekerle & Kollar, 2022). Furthermore, significant differences in the integration of technology through different learning modes were observed between teachers from different countries, which is most likely a consequence of the different educational contexts in which teachers work. This needs further investigation.

#### Differences in TT by country

In addition, the analysis of descriptive statistics showed that Austrian and Montenegrin contexts most often used active TT, while Turkish, Croatian, Republic of Serbia, Czech Republic, and Slovenian contexts most often used passive TT. Generally, through the analysis of results from all countries, it can be observed that passive TTs are most often used. In previous research on this topic, it was also observed that in Swiss (Antonietti et al., 2023a) and German (Fütterer et al., 2023a) contexts are most often used passive TT (e.g. word-processing software is used the most). Furthermore, significant differences in using different TT were observed between teachers from different countries, which is also most likely a consequence of the different educational contexts in which teachers work. This needs further investigation.

### Relationships between TT and TI into ICAP learning/engaging modes

*The 3rd part*: A significant and positive relationship exists between passive TT and TI into passive, active, constructive, and interactive learning mode. However, this correlation between passive TT and TI into passive learning mode is high compared to others while between passive TT and TI into interactive learning mode is relatively low (the effect size values show the same). We can notice that teachers who use internet learning platforms, games, online tests and quizzes, specific-subject learning software, online research software and word-processing software, integrate technology into teaching mostly by giving students a passive role, then an active one, constructive, and at least an interactive role. The similar was observed in research (Antonietti et al., 2023a; Fütterer et al., 2023a).

In addition, a significant and positive correlation exists between active TT and TI into active, constructive, and interactive learning mode. Also, a negative correlation was observed between active TT and TI into passive learning mode (small effect size). However, this correlation between active TT and TI into interactive learning mode is relatively high compared to others while correlation between active TT and TI into passive learning mode is relatively low (effect size values show the same). We can notice that teachers who use online communication software, spreadsheet and calculation software, student work presentation/publication software, learning management system and video recording and editing software integrate technology into teaching mostly by giving students an interactive role, then a constructive, active, and the least passive role. Similar was observed in previous research (Antonietti et al., 2023a; Fütterer et al., 2023a).

To summarize, teachers' uses of active or passive TTs affects their TI into different ICAP learning/engaging modes. Passive TT predict TI into passive, active, and constructive learning modes more, while active TT predict TI in interactive, constructive and active learning modes more. Teachers' use of passive TT indicates that they integrate technology the most into passive learning mode, while their use of active TT indicates that they integrate technology the most into interactive learning mode. The relationship between different types of technologies and ICAP learning modes should be further explored, but by expanding the list of technology activities and examining their individual relationship with each of the ICAP learning modes to identify those technology activities most suitable for integration into constructive and interactive learning mode.

## Contributions, limitations, and recommendations for future research

Successful integration of technology does not mean only technical equipment of institutions, the inclusion of technology in teaching practice, and its frequent usage. The quality of integration depends on the way it is used to engage students cognitively as much as possible (Antonietti et al., 2023a; Anđić et al., 2022; Deepika et al., 2022; Janković et al., 2023; Wekerle et al., 2022). Cognitive engagement depends on the type of activity to which students are exposed. On the scale from passive to interactive, the most cognitively engaged students are those who are involved in interactive activities supported by technology. The transition from passive to active use of technology can be achieved through activities that go beyond the level of pure viewing of teaching material and receiving information through technology i.e. in the following ways:

o Through the design and creation of activities, that engage students to manipulate technology, or through technology to actively observe what they are investigating (activating the processes of storing, activating, and linking);
o Through designing and creating activities that engage students to use technology constructively, i.e. as learning material that will enable them to generate their insights and ideas (activating the processes of storing, activating, linking, and inferring—infer-from-own);
o Through designing and creating activities that engage students to use technology collaboratively, i.e. as learning material in cooperation with peers, which will enable them to create innovative knowledge (activating the processes of storing, activating, linking, inferring—infer-from-own, and infer-from-other).

In addition to providing insights into strategies for transitioning from passive to active TI relevant to educator-practitioners, our study offers concrete contributions to the research community. It is characterized by certain limitations based on which recommendations for future studies within this issue arise. We will list them in the text below.

### Contributions

Compared to previous studies, the contribution of our study is reflected in:

o Providing significant insights into the reliability and validity of the newly created instrument—ICAP-TS for the assessment of TI by teachers at the international level;
o Classification of the most commonly used TT in education into categories (passive and active), which most closely describe them;
o Determining the way of TI through different ICAP modes, as well as its frequency on a diverse sample of teachers;
o Discovering the differences between the way technology is integrated and its frequency, as well as the differences between the usage of passive and active technologies by teachers from different educational contexts at the international level;
o Determining the relationships between different TT and ICAP learning modes on a diverse sample of teachers;
o Gaining significant insights into which TT predicts which ICAP modes in learning at the international level.

### Limitations

In addition to the contributions, our study is characterized by certain limitations, which are as follows:

o Although the study is international, not all European countries (nor from the wider region) are covered, which partially limits the generalization of the results on a more global level;
o The limitation of the generalization of the results on a more global level is also reflected in their application both in certain parts of Europe and outside Europe;
o This is, for example, reflected in the following: Gaining insight into the ways of integrating technology, its frequency, and the most commonly used TT provides the opportunity for developers, policymakers, stakeholders, and practitioners to find problems—issues with the quality of TI, as well as to direct them towards the elimination of these issues to reorganize teaching practice and improve it. This can only be done in the countries where the research was conducted; By discovering the relationships between different TT and TI in different ICAP modes, the possibility of predicting teaching practice is revealed (active TT predicts more active ICAP modes, passive TT—more passive modes), which also provides a chance for its reorganization and improvement in terms of higher quality TI in the educational contexts of the countries where the research was carried out. The educational contexts of different countries are conditioned by many factors such as culture, tradition, society, politics, and economic status vary widely. For the applicability of the results to be possible at the more global level, it is necessary to take into account, consider, and include different educational contexts in the research and gain insight into a unique picture of technology integration, which will provide a basis for further work and possible guidelines on improving educational practice regarding this aspect.

### Recommendations for future research

ICAP-TS is a relatively newly developed instrument representing an essential literature need. Considering that research in this area is still fresh, the following recommendations are also stated:

o Our study shows that the Active1 item predicts the Active sub-scale at a relatively critical level compared to the others. Similarly, the Passive1 item predicts the Passive sub-scale at a relatively more critical level than the others. Reviewing or revising these two items may be among the subjects of future studies;
o In addition, the active sub-scale’s internal consistency, reliability, and convergent validity values are critical for Czech Republic. The results of this study can be compared by repeating the research on another sample in this country;
o Discriminant validity values for constructive and active sub-scale are at critical thresholds for all countries except Czech Republic. Future research topics can include reviewing, revising, or adding new items to the ICAP-TS related to ICAP theory;
o In this study, we explored the relationships between technology types and ICAP modes. Future studies should go further and examine the relationships between ICAP sub-scales to determine how teachers' TI in different learning modes predicts each other;
o The topic of future studies can be the creation of a unique model through the combination of the ICAP theory with other theories (such as the Technology acceptance model, Will, skill, and tool model, Cognitive load theory model Maričić et al., 2023, etc.) with the aim of a deeper analysis within this issue and examination of the mutual connection of these constructs. This would provide fresh and significant insights into the integration of technology viewed from different perspectives;
o In addition to the above it is also recommended to extend this study over time to see how technology integration evolves with targeted educational interventions.

Finally, we would like to emphasize that the model proposed in this study could be one of many theoretically viable models.

## Data Availability

All data and materials as well as software application or custom code support published claims and comply with field standards. The data generated during and/or analyzed during the current study are available from the corresponding author on reasonable request.

## Appendix

See Tables 4, 5, 6, 7, 8, 9, 10, 11, 12, and 13.

**Table 4:** Observed values of first-order confirmatory factor analysis of ICAP-TS by country

	AT	CZ	HR	ME	RS	SI	TR	All
ChiSqr	119.282	139.768	151.059	219.149	253.025	126.378	112.090	623.178
*df*	48.000	48.000	48.000	48.000	48.000	48.000	48.000	48.000
*p*	.000	.000	.000	.000	.000	.000	.000	.000
ChiSqr/df	2.485	2.912	3.147	4.566	5.271	2.633	2.335	12.983
GFI	.938	.946	.894	.811	0.948	0.914	.920	.957
SRMR	.055	.047	.057	.064	.039	.042	.040	.042
CFI	.960	.952	.943	.901	.976	.956	.975	.969

*AT* Austria, *CZ* Czech Republic, *HR* Croatia, *ME* Montenegro, *RS* Republic of Serbia, *SI* Slovenia, *TR* Turkey

**Table 5:** Construct reliability and validity values for ICAP-TS by country

Countries	ICAP	Cronbach's Alpha (standardized)	Cronbach's Alpha (unstandardized)	CR (rho_c)	AVE
AT	Active	.806	.806	.806	.582
	Constructive	.870	.869	.870	.689
	Interactive	.722	.720	.735	.482
	Passive	.762	.749	.768	.572
CZ	Active	.675	.653	.672	.460
	Constructive	.760	.760	.763	.519
	Interactive	.804	.803	.803	.578
	Passive	.806	.794	.812	.598
HR	Active	.794	.778	.792	.609
	Constructive	.875	.874	.877	.703
	Interactive	.918	.916	.918	.789
	Passive	.832	.824	.834	.644
ME	Active	.894	.893	.894	.740
	Constructive	.935	.935	.939	.835
	Interactive	.727	.726	.677	.431
	Passive	.749	.749	.768	.529
RS	Active	.861	.860	.870	.697
	Constructive	.920	.920	.920	.794
	Interactive	.924	.923	.923	.800
	Passive	.884	.881	.884	.733
SI	Active	.811	.805	.802	.602
	Constructive	.877	.877	.877	.703
	Interactive	.915	.915	.919	.789
	Passive	.850	.846	.849	.671
TR	Active	.886	.884	.889	.738
	Constructive	.931	.931	.932	.820
	Interactive	.948	.948	.948	.858
	Passive	.914	.914	.918	.786
All	Active	.811	.809	.820	.618
	Constructive	.897	.897	.898	.745
	Interactive	.877	.877	.877	.704
	Passive	.841	.838	.844	.659

*AT* Austria, *CZ* Czech Republic, *HR* Croatia, *ME* Montenegro, *RS* Republic of Serbia, *SI* Slovenia, *TR* Turkey

**Table 6:** Descriptive statistics for different TT

Educational technologies		*M*	*SD*	Skewness	Kurtosis
EdTech1	Specific-subject learning software	3.17	1.296	− .110	− 1.056
EdTech2	Online tests and quizzes	2.60	1.224	.287	− .820
EdTech3	Word-processing software	2.85	1.280	.116	− 1.030
EdTech4	Spreadsheet and calculation software	2.51	1.296	.402	− .958
EdTech5	Games	2.63	1.271	.272	− .979
EdTech6	Internet learning platform	2.94	1.313	.024	− 1.097
EdTech7	Drawing and image editing software	2.59	1.302	.320	− 1.014
EdTech8	Video recording and editing software	2.12	1.199	.837	− .311
EdTech9	Online research software	3.42	1.216	− .328	− .811
EdTech10	Online communication software	2.37	1.323	.590	− .840
EdTech11	Students' work presentation/publication software	2.93	1.206	.083	− .897
EdTech12	Learning management system	2.66	1.312	.311	− 1.024

**Table 7:** Factor loads for different TT

Educational technologies	Component	
	1	2
Internet learning platform	.818	
Games	.775	
Online tests and quizzes	.716	
Specific-subject learning software	.668	
Online research software	.666	
Word-processing software	.604	
Online communication software		.782
Drawing and image editing software		.773
Spreadsheet and calculation software		.736
Students work presentation/publication software		.645
Learning management system		.622
Video recording and editing software		.600

**Table 8:** Descriptive statistics for ICAP-TS by country

	ICAP	*M*	*SD*	Skewness	Kurtosis
AT	Interactive	9.51	2.907	− 0.322	− 0.403
	Constructive	6.62	3.099	.734	− 0.099
	Active	7.82	2.962	.310	− 0.421
	Passive	8.07	2.960	.427	− 0.261
CZ	Interactive	7.75	2.771	.075	− 0.567
	Constructive	8.38	2.746	.041	− 0.501
	Active	9.45	2.506	− 0.347	.268
	Passive	10.86	2.577	− 0.370	− 0.010
HR	Interactive	9.57	3.532	− 0.498	− 0.579
	Constructive	10.52	3.070	− 0.745	.257
	Active	10.01	3.080	− 0.581	− 0.145
	Passive	11.27	2.855	− 0.532	− 0.122
ME	Interactive	8.11	2.810	.221	− 0.210
	Constructive	8.38	3.489	.314	− 0.716
	Active	8.32	3.173	.252	− 0.545
	Passive	8.30	2.751	.477	.231
RS	Interactive	8.36	3.541	.171	− 0.975
	Constructive	9.01	3.522	− 0.049	− 0.995
	Active	8.45	3.338	.189	− 0.857
	Passive	10.82	3.167	− 0.505	− 0.505
SI	Interactive	8.00	2.920	.207	− 0.417
	Constructive	8.56	2.773	.164	− 0.361
	Active	8.31	2.805	.394	.060
	Passive	11.21	2.824	− 0.367	− 0.432
TR	Interactive	9.48	3.665	− 0.238	− 0.956
	Constructive	9.77	3.675	− 0.301	− 0.987
	Active	9.30	3.455	− 0.065	− 0.949
	Passive	11.74	2.658	− 0.595	− 0.297

*AT* Austria, *CZ* Czech Republic, *HR* Croatia, *ME* Montenegro, *RS* Republic of Serbia, *SI* Slovenia, *TR* Turkey

**Table 9:** ANOVA analysis for ICAP learning modes by country

	Source of variances	Sum of squares	*df*	Mean square	Welch's t-test	*p*	Difference
Interactive	Between Groups	1053.69	6.00	175.62	18.21	.000	AT-CZ, AT-ME, AT-RS, AT-SI CZ-RS HR-CZ HR-ME, HR-RS, HR-SI TR-CZ TR-ME, TR-RS, TR-SI
	Within Groups	23,877.61	2270.00	10.52			
	Total	24,931.31	2276.00				
Constructive	Between Groups	2333.52	6.00	388.92	39.15	.000	AT-All countries HR-CZ, HR-ME, HR-RS, HR-SI, HR-TR RS-CZ TR-CZ, TR-ME, TR-SI
	Within Groups	23,923.95	2270.00	10.54			
	Total	26,257.47	2276.00				
Active	Between Groups	993.74	6.00	165.62	19.13	.000	CZ-AT, CZ-ME, CZ-RS, CZ-SI HR-AT, HR-ME, HR-RS, HR-SI TR-AT, TR-RS, TR-SI
	Within Groups	21,565.52	2270.00	9.50			
	Total	22,559.26	2276.00				
Passive	Between Groups	3168.76	6.00	528.13	64.32	.000	CZ-AT, CZ-ME HR-AT, HR-ME RS-AT RS-ME SI-AT, SI-ME TR-AT, TR-CZ, TR-ME, TR-RS
	Within Groups	19,173.20	2270.00	8.45			
	Total	22,341.96	2276.00				

*AT* Austria, *CZ* Czech Republic, *HR* Croatia, *ME* Montenegro, *RS* Republic of Serbia, *SI* Slovenia, *TR* Turkey

**Table 10:** Descriptive statistics for different TT by country

Country	TT type	*M*	*SD*	Skewness	Kurtosis
AT	Passive TT	12.97	4.68	.950	1.187
	Active TT	17.70	3.76	.215	− 0.174
CZ	Passive TT	17.16	4.32	− 0.104	− 0.047
	Active TT	12.16	4.12	.813	.943
HR	Passive TT	20.02	5.67	− 0.423	− 0.499
	Active TT	15.99	6.05	.351	− 0.742
ME	Passive TT	14.83	5.87	.711	.125
	Active TT	15.52	4.16	.753	.382
RS	Passive TT	18.78	5.61	.046	− 0.550
	Active TT	14.92	6.04	.609	− 0.292
SI	Passive TT	16.53	4.99	.216	− 0.443
	Active TT	13.76	4.87	.784	.570
TR	Passive TT	20.83	5.7	− 0.303	− 0.633
	Active TT	18.7	6.49	.078	− 0.954

*AT* Austria, *CZ* Czech Republic, *HR* Croatia, *ME* Montenegro, *RS* Republic of Serbia, *SI* Slovenia, *TR* Turkey

**Table 11:** ANOVA analysis for different TT by country

	Source of variances	Sum of Squares	*df*	Mean Square	Welch's t-test	*p*	Difference
Passive TT	Between Groups	11,881 .15	6	1980 .19	74.40	.000	AT-All countries HR-CZ, HR-ME, HR-SI RS-CZ, RS-ME, RS-SI TR-CZ, TR-ME, TR-RS, TR-SI
	Within Groups	61,758 .11	2219	27 .83			
	Total	73,639 .26	2225				
Active TT	Between Groups	8843 .69	6	1473 .94	71.63	.000	AT-CZ, AT-HR, AT-ME, AT-RS, AT-SI CZ-All countries SI-HR, SI-ME TR-HR, TR-ME, TR-RS, TR-SI
	Within Groups	62,825 .69	2227	28 .21			
	Total	71,669 .38	2233				

*AT* Austria, *CZ* Czech Republic, *HR* Croatia, *ME* Montenegro, *RS* Republic of Serbia, *SI* Slovenia, *TR* Turkey

**Table 12:** Discriminant validity—Heterotrait-monotrait ratio (HTMT) matrix

	Active	Active TT	Constructive	Interactive	Passive	Passive TT
Active						
Active TT	.524					
Constructive	.815	.535				
Interactive	.734	.654	.791			
Passive	.655	.408	.597	.478		
Passive TT	.639	.749	.633	.536	.697

**Table 13:** Predictive power values of the model

	*R*^2^	*Q*^2^
Active	.308	.298
Active TT	.407	.406
Constructive	.329	.308
Interactive	.333	.216
Passive	.350	.347
